# Effect of an enzyme-containing mouthwash on the dental biofilm and salivary microbiome in patients with fixed orthodontic appliances: a randomized placebo-controlled pilot trial

**DOI:** 10.1093/ejo/cjac062

**Published:** 2022-10-10

**Authors:** Tove Hoffstedt, Lea Benedicte Skov Hansen, Svante Twetman, Mikael Sonesson

**Affiliations:** Orthodontic clinic, public dental health, Karlshamn, Region Blekinge, Sweden; Novozymes A/S, Bagsværd, Denmark; Department of Odontology, Faculty of Health and Medical Sciences, University of Copenhagen, Denmark; Department of Orthodontics, Faculty of Odontology, Malmö University, Sweden

## Abstract

**Background:**

Mouthwashes containing oral antiseptics or enzymes are suggested suitable for controlling biofilm accumulation in patients with fixed appliances and thereby limiting unwanted side effects during the orthodontic treatment.

**Objectives:**

To evaluate the effect of an enzyme-based mouthwash on the amount of dental biofilm and the composition of the salivary microbiome in patients undergoing treatment with fixed orthodontic appliances.

**Trial design:**

Randomized double-blind placebo-controlled trial.

**Material and methods:**

In total, 35 young adolescents (14–18 years) under treatment with fixed appliances were consecutively enrolled and randomly allocated to an experimental or a placebo group by opening a computer-generated numbered envelope. The subjects were instructed to rinse twice daily during an intervention period of 8 days with experimental mouthwash or placebo without active enzymes. Unstimulated whole saliva samples were collected at baseline and after 8 days. The participants and examiner were blinded for the allocation. The primary outcome was the Orthodontic Plaque Index (OPI) and the secondary was the composition of the salivary microbiome.

**Results:**

In total, 28 adolescents (21 females and 7 males) completed the trial and there were no differences in age, clinical, or microbial findings between the test (*n* = 14) and the placebo group (*n* = 14) at baseline. We found a decreased OPI in the test group after 8 days and the difference was statistically significant compared with the placebo group (*P* < 0.05). There were no significant treatment effects on the richness and global composition of the salivary microbiome.

**Harms:**

In total, one participant in the test group claimed nausea and abandoned the project. In total, two participants did not like the taste of the mouthwash but used it as instructed. No other adverse events or side effects were reported.

**Limitations:**

Short-term pilot trials may by nature be sensitive for selection and performance biases and are not designed to unveil persisting effects.

**Conclusion:**

Daily use of enzyme-containing mouthwash reduced the amount of dental biofilm in adolescents under treatment with the fixed orthodontic appliances, without affecting the composition of the salivary microbiota.

**Ethical approval:**

Approved by the Regional Ethical Board, Lund, Sweden (Dnr 2020-05221).

**Clinical Trial Registration:**

NCT05033015.

## Introduction

Caries is a biofilm-mediated disease and public health problem with social and economic consequences for individuals, communities, and countries (1). The disease is a result of a sugar-driven, complex interaction between the commensal microbiota, host susceptibility, and environmental factors. The dental biofilm (dental plaque) is an aggregated cluster of microorganisms in a polysaccharide matrix in which cells adhere to each other on a tooth surface that is protected. The resident oral microbiota is diverse, natural, and beneficial to the host when symbiosis exists. Environmental perturbations can, however, shift the composition to a dysbiotic state, preceding diseases such as caries, periodontitis, and its sequelae (2). The main drivers of such caries-associated dysbiosis are long-term low-pH conditions, induced by excessive and frequent intake of sugars, and non-regular mechanical cleaning (2, 3).

For many decades, caries was regarded as an infectious transmissible disease with *Streptococcus mutans* named the ‘arch-criminal’ (4). Consequently, clinical strategies to suppress the mutans streptococci load in the oral cavity with topical antibacterial substances and anti-adhesive strategies were advocated (5). The ‘one pathogen, one disease’ paradigm is now replaced by a holistic concept with clusters of the microbial community as an entity of pathogenicity (4). The current understanding is that the dental biofilm should be controlled rather than eradicated and research is presently focused on a gentle disruption with products that support and maintain a health-associated oral microbiome (2, 6).

Treatment with fixed orthodontic appliances is associated with impaired oral hygiene and dysbiotic conditions adjacent to the bracket base (7, 8). This may result in demineralizations of the enamel, often called white spot lesions (WSLs) that might jeopardize the aesthetic outcome of the treatment (9). The prevalence of WSL is fairly high (30–50%) among adolescents and self-applied fluorides cannot completely prevent such lesions (10). It is therefore important to adopt novel approaches to reduce and combat these unwanted adverse effects. A systematic review has shown evidence of the effectiveness of oral mouthwashes containing various antiseptics in the control of cariogenic biofilm in patients with fixed orthodontic appliances (11). Another approach is the addition of enzymes to mouthwashes that break down large polysaccharides in the oral biofilm to smaller, more soluble polysaccharides that are easier to remove (12). The aim of the present pilot study was to evaluate the effect of an experimental mouthwash containing a blend of natural enzymes, on the amount of dental biofilm and composition of the salivary microbiome in patients undergoing treatment with fixed orthodontic appliances. The null hypothesis was that neither the amount of biofilm adjacent to the brackets nor the composition of the salivary microbiome would differ from that of a placebo mouthwash without active enzymes.

## Materials and methods

### Study design, ethical approval, and registration

The pilot study employed a randomized, double-blind placebo-controlled design with two parallel arms. The project was approved by the Swedish ethical review authority, Sweden (Dnr 2020-05221) and registered in Clinical Trials.gov Identifier (NCT05033015).

### Subjects

We invited 60 eligible patients (14–18 years) at the Orthodontic Specialist Clinic in Karlshamn, Sweden to enter the study. The subjects were consecutively enrolled and the inclusion criteria were: 1. being under treatment with fixed orthodontic appliances (uni- or bi-maxillary) for at least 3–6 months and 2. visible biofilm accumulation around the bracket bases of at least six teeth. Patients with caries lesions, periodontal disease, and/or soft tissue pathology were not invited. Likewise, we excluded patients with known food allergies or a history of allergies to ingredients in the test product, including allergy against enzymes. In total, 35 patients accepted the invitation and obtained verbal and written information about the project. We collected a signed consent from each patient and the custodians. The subjects were randomly allocated to the test or placebo group after the baseline registrations by opening a computer-generated numbered envelope.

### Intervention and test products

We provided all the subjects with a standardized enzyme-free toothpaste with 1450 ppm sodium fluoride to be used twice daily during the course of the study. After the baseline clinical registration, we asked the participants to rinse their mouth thoroughly by swishing 10 ml of the assigned liquid around the teeth morning and evening for 30 seconds. After rinsing, the subjects should spit out, avoid water rinsing, and most importantly, we requested them to separate tooth brushing from mouth washing by at least 30 min. The duration of the intervention was 8 days. The participants were instructed to return the dispensed mouthwash tubes to the clinic at the next visit. The experimental mouthwash contained an enzyme blend of DNase, mutanase, and beta-glucanase (BioFresh^®^ Clean, Novozymes A/S) in an aqueous solution as shown in Table 1. The placebo mouthwash had an identical taste and colour but contained no active enzymes. To secure the blinding of both the investigator and the participants, the products came in coded tubes, each containing exactly 10 ml of the product. The clinical investigator sent a twice-daily SMS (morning and evening) reminder (Servicewell, Lund, Sweden) to the patients in order to enhance compliance. An independent university-based monitor guaranteed allocation concealment.

**Table 1.** Content of the experimental mouthwash.

Ingredient	Concentration in the sample % (w/w)
Enzyme mix: • Enzyme A: DNAse • Enzyme B: Mutanase • Enzyme C: Beta-glucanase	60 ppm of each enzyme [0.006%]
Sorbitol	40.0%
Sodium citrate	0.45%
Monobasic—sodium phosphate	1.04%
Potassium sorbate	0.20%
Mint flavour	0.10%
Water	58.21%

### Clinical registrations and endpoints

The primary outcome was the amount of dental biofilm around the orthodontic brackets and one principal examiner (TH) performed the registrations at baseline and after 8 days. To visualize the biofilm, teeth were illuminated with a violet UV light in detection mode (D-Light Pro, GC Europe). The biofilm was then scored according to Beberhold et al. (13); score 0 = brackets are plaque-free; score 1 = isolated plaque islands on one tooth surface at the bracket base; score 2 = plaque on two tooth surfaces at the bracket base; score 3 = plaque on three tooth surfaces at the bracket base; and score 4 = plaque on all tooth surfaces at the bracket base and/or gingival inflammation. The buccal surfaces of the upper and lower anterior teeth (incisors and cuspids) and premolars were registered. We divided the total sum of the scores with the number of teeth to express the Orthodontic Plaque Index (OPI) at the patient level. The examiner also scored the buccal mucosa along the gingival margin as 0 = normal gingiva (bleak and firm), or 1 = presence of gingivitis (clinical visible registration). To evaluate the inter-examiner reliability of the OPI, two examiners scored independently the biofilm at the bracket base of totally 178 teeth on 10 teenagers under treatment with fixed orthodontic appliances, not taking part in the pilot study.

### Saliva sampling and analyses

The examiner collected unstimulated whole saliva samples at baseline and after 8 days before the biofilm scoring. The subjects rinsed with tap water, accumulated saliva in their mouth and expectorated in a capped plastic tube (approximately 1.0 ml). The tube was immediately frozen and stored at −18°C until transportation and further processing at the Novozymes A/S laboratory in Denmark. We included negative control samples (no material/DNA) as well as positive controls consisting of a known mock community to validate the analyses. The DNA extraction was performed with Macherey-Nagel™ NucleoSpin™ Soil kit and the V3–V4 region of 16S rRNA gene was PCR amplified using universal primers. The amplicons were sequenced using Illumina MiSeq 300 bp paired end. We provide a full description in Supplementary Appendix S1.

### Side effects

All the possible subjectively perceived side effects during the intervention were immediately reported to the clinical investigator and the subjects could discontinue their participation at any time without further motivation.

### Power calculation and statistical methods for clinical data

Due to the lack of previous clinical data with respect to the experimental mouth rinse, we anticipated that a mean difference of 0.5 OPI units (SD 0.4) would be clinically meaningful. A calculation with *α* = 0.05 and *β* = 0.8 indicated that 18 patients in each group should be enrolled in this pilot project. We processed the clinical data with the IBM–SPSS software (version 27.0, Chicago, USA). For continues data, differences between groups on the subject level were compared with unpaired *t*-tests. We tested the relative within-group differences with an N-1 chi-square test. Inter-examiner reliability was calculated and expressed with the Cohen’s kappa value. A *P*-value less than 0.05 was regarded as statistically significant. We performed the statistical calculations before the group allocation was unveiled.

### Bioinformatics and statistical analysis of the saliva microbiome samples

A complete description is provided in Supplementary Appendix S1. We used the USEARCH pipeline to generate zero radius operational taxonomic units (OTUs) (14). To account for varying in sequencing depths the samples were normalized by rarefying down to 10 000 reads. For the global composition of the salivary microbiome at baseline, we applied the Adonis Permanova test on the dissimilarity matrix using the Bray–Curtis metric (15). To investigate if the mouthwash introduced a change in the global microbiome composition over time, the Bray–Curtis dissimilarity between baseline and follow-up samples for each individual was tested using a paired *t*-test. We established a linear mixed model to test the effects of the mouthwash compared with the placebo group on microbial richness from baseline to follow-up. The null hypothesis was that the change in richness between baseline and follow-up for the test group was equal to the change in the placebo group. We used a similar model to test for significant changes for all the OTUs present in more than 32 samples, where each OTU count was log transformed. We corrected all *P*-values for multiple testing according to Benjamini and Hochberg (16).

## Results

### Baseline data

**Figure 1.**
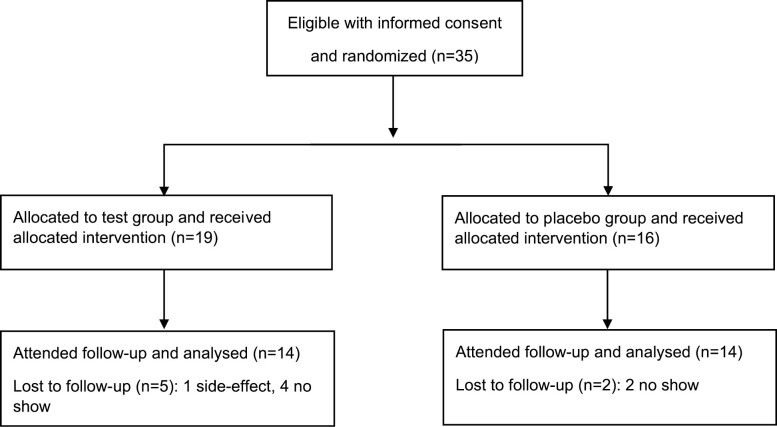
Flow chart of the trial according to CONSORT 2010.

The mean age was 15.4 years in the test group and 16.8 years in the placebo group. The most common orthodontic treatment indication was moderate-to-severe crowded dental arches and 18 patients had 2–4 premolars extracted. All the patients had pre-adjusted fixed orthodontic appliances (0.022 slot size, MBT prescription, 3M™ Victory Series™ Low Profile Brackets, Unitek, CA, USA) in the maxilla and 22 were under treatment with bi-maxillary braces. In total, three patients in each group were bonded just in the maxilla. The trial was performed during the period between February 2021 and May 2021. For various Covid-19-related reasons, only 28 subjects (21 females and 7 males) completed the study protocol, giving a dropout rate of 18%. A flow chart is presented in Figure 1.

### Clinical findings

**Figure 2.**
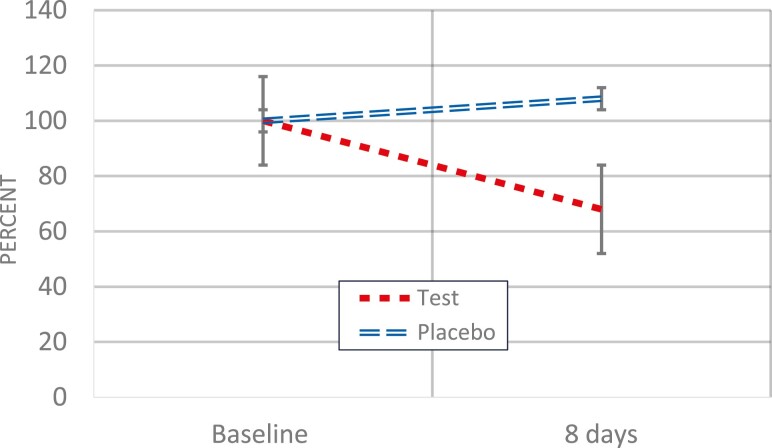
Relative change in orthodontic plaque index (OPI) scores from baseline to follow-up in the test and placebo groups. The vertical bars denote the standard error. The decrease in the test group was statistically significant (*P* < 0.05).

Subjects in the placebo group had a slightly higher mean OPI value at baseline but the difference was not statistically significant compared with the test group (Table 2). After 8 days, a decrease was noted in the mouthwash test group but not in the placebo group (*P* < 0.05). The relative difference was 38% as illustrated in Figure 2. In total, six patients in the test groups and nine patients in the placebo group displayed clinical visible gingivitis at baseline. The corresponding numbers after 8 days were 3 and 9 patients, respectively. The inter-examiner test showed a 78% agreement of the OPI scores. The Cohen’s-weighted Kappa was 0.734 (standard error 0.039), indicating a good agreement.

**Table 2.** Orthodontic plaque index (OPI) on subject level (*n*) at baseline and the 8-day follow-up. Values in the table denote mean and standard deviations.

Time	*n*	Test OPI	*n*	Placebo OPI	*t*-value	*P*
Baseline	19	0.72 (0.70)	16	1.12 (0.70)	−1.69	NS
8-Day follow-up	14	0.49 (0.51)	14	1.21 (0.74)	−2.98	<0.05

Differences were tested with an unpaired two-sided *t*-test.

### Microbial findings

**Figure 3.**
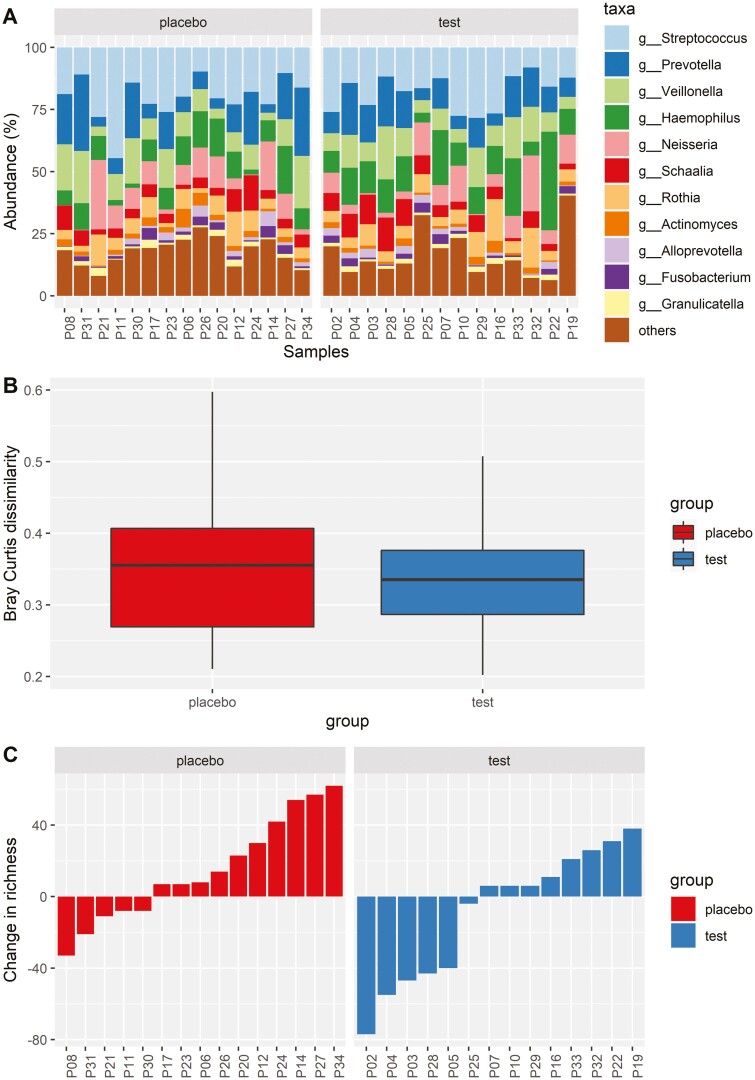
(A) Stacked bar plot showing the relative abundance in the percentage of the 11 most abundant genera for each baseline sample (*n* = 29). The ‘other’ category contains all the lower abundant genera. (B) Boxplot showing the pairwise Bray–Curtis dissimilarity between baseline and follow-up sample for the placebo (red) and test (blue) group, with no statistical difference between groups (*P* = 0.704). (C) Barplot showing the change in richness from baseline to follow-up for each subject. We measured the richness by counting the number of unique operational taxonomic units (OTUs) in each sample and the subjects are visualized according to the placebo (red) and test (blue) groups. No statistically significant change was found between the groups, but there was a trend toward an increased richness in the placebo group compared with the test group from baseline to follow-up (*P* = 0.06).

**Figure 4.**
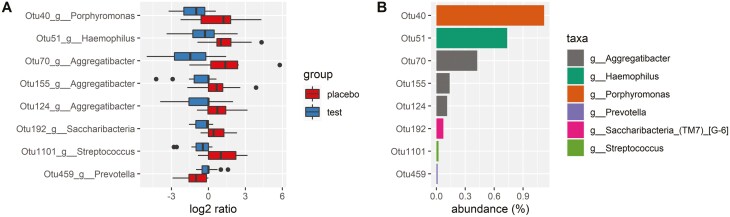
(A) Boxplot showing the log2 ratio of the relative abundance of the eight significantly different OTUs between baseline and follow-up. The red box shows the change in abundance for the placebo group and the blue box shows the change for the test group. (B) Barplot showing the relative abundance for the eight significantly different OTUs in the percentage of all the samples. The bars are coloured according to taxonomic classification on genus level.

We display the baseline composition of the salivary microbiome at Genus level and the most abundant taxa in Figure 3A. There was no difference in baseline microbial composition between the test and placebo groups (*P* = 0.317) and we found a consistent composition across time, with no statistical differences between the groups in the pairwise comparison of the global microbial composition at baseline and follow-up (Figure 3B). There were no significant treatment effects on the microbial richness although we observed a tendency towards an increase in the placebo group compared with the test group (*P* = 0.06; Figure 4A). In total, nine bacterial species seemed affected by the treatment (Figure 4B). Most consistent was the finding of a relative increase in abundance of three *Aggregatibacter* species in the placebo group.

### Side effects

One participant in the test group claimed nausea and ‘weird taste’ and abandoned the project after the first day. Two participants did not like the taste of the mouthwash but used it as instructed. No other adverse events, or side effects, were reported to the investigators. All the participants except one returned all the dispensed mouth rinse tubes to the clinic after eight days as agreed.

## Discussion

Treatment with fixed orthodontic appliances interferes with mechanical tooth cleaning which results in the accumulation of dental biofilm, in particular, among patients with irregular tooth-brushing habits. Our intention with this pilot study was, therefore, to evaluate the possible effects of the enzyme-based mouthwash on a mature dental biofilm rather than on the ‘de novo’ formation. In this context, it is important to keep in mind that a biofilm with a climax bacterial community may be harder to affect with a mouthwash than a regularly disrupted dental biofilm. Therefore, our results with respect to the primary endpoint was encouraging. We found that the experimental enzyme-containing product could reduce the biofilm levels in adolescents under treatment with fixed orthodontic appliance and therefore, we rejected the null hypothesis. The mean difference between the groups was 0.7 OPI units, which was of clinical importance. The mode of action for the tested blend of enzymes (DNAse, mutanase, and glucanase) was most likely disintegrating the architecture of the biofilm through hydrolysis of extracellular DNA in the biofilm matrix, create a change in the diffusion properties, and break down large bacteria-produced polysaccharides via hydrolysis (17). In addition, mutanase has the ability to affect the synthesis and structure of sticky, extracellular glucans in dental biofilm (18). The prevalence of gingivitis was not stated as a measurable outcome, but was evaluated as a clinical finding. No differences in gingivitis between the groups was seen, which was not surprising considering its dual etiology. Gingivitis normally reflects a low-grade inflammation due to excessive biofilm accumulation but may also appear through mechanical irritation from the orthodontic appliance. The clinical data obtained from this pilot study can be useful for the sample size calculations of future larger and extended clinical studies with enzyme-based mouthwashes.

Concerning the secondary outcome, it was important to rule out any negative impact on the quality of the oral microbiota. The analyses of the salivary microbiome displayed a typical composition for the human oral environment on Phylum, Family, and Genus levels. We found no major treatment effects on the composition which was in line with previous studies on short-term interventions with agents such as fluoride, xylitol, and probiotics (19–21). This indicated that the experimental mouthwash did not affect the dental biofilm in a detrimental way and thus, the microbial null hypothesis was accepted. The significance of the three abundant *Aggregatibacter* species in the placebo group is unclear but most likely a finding by chance. This genus contains both friendly and pathogenic strains and such a shift would have been notable if it appeared in the test group. Once established, the oral microbiome is rather stable and only significantly affected by the long-term perturbations of the oral ecosystem (19) but further research with enzyme-based mouthwashes should extend the duration of the intervention to unveil any possible translational effects on the microbiota during orthodontic treatment. From this pilot study we, have established a base for future sample size calculations and gained information and experience on the feasibility of the present mouthwash.

### Strengths and limitations

The strength of this project was the robust randomized double-blinded placebo-controlled design and the clinical scoring was robust and reliable. The efficacy of tooth brushing has an effect on biofilm formation. However, the two groups were under orthodontic treatment for at least 3 months before the start of the trial and the baseline levels in the groups are similar. Thus, it is assumable that tooth-brushing routines were the same for the test and placebo groups. Nevertheless, there were still some uncertainties to consider. We experienced an unexpected high-attrition rate due to COVID-19-related restrictions, which unfortunately lowered the power of the study. We sampled and analysed unstimulated whole saliva as a proxy for the oral microbiome and dental biofilm. The salivary microbiota constitutes a compilation of bacteria shed from all oral surfaces, including the tongue and the throat (22, 23). However, as saliva harbours a less diverse community than the dental biofilm (24), the present findings may not fully reflect the microbial composition of biofilm samples collected directly from the orthodontic appliances. We also failed to monitor the compliance rigorously but we have reasons to believe, in part because of the SMS reminders and the number of returned dispensed mouthwash tubes, that the subjects completing this pilot study of 8 days were highly motivated and used the mouthwash per protocol. An interesting observation was that no ‘placebo-effect’ was evident in the placebo group, a phenomenon commonly reported in connection with double-blinded trials.

## Conclusion

A twice-daily use of an enzyme-containing mouthwash decreased the amount of dental biofilm in adolescents with fixed orthodontic appliances over an 8-day period without significantly affecting the composition of the salivary microbiome. The results of this pilot study indicate that the mouthwash, when applied in adjunct to regular tooth brushing, may be beneficial for adolescent orthodontic patients and justify a larger clinical trial with the prolonged duration.

## Supplementary Material

cjac062_suppl_Supplementary_Appendix_S1

## Data Availability

The data underlying this article were provided by Region Blekinge under license/by permission. Data will be shared on request to the corresponding author with permission of Region Blekinge.
