# Supplementary material for: Effect of an enzyme-containing mouthwash on the dental biofilm and salivary microbiome in patients with fixed orthodontic appliances: a randomized placebo-controlled pilot trial
Source: Eur J Orthod. 2022 Oct 10;45(1):96–102. doi: 10.1093/ejo/cjac062 (PMC9912700; doi:10.1093/ejo/cjac062)
Supplement: cjac062_suppl_Supplementary_Appendix_S1 [file cjac062_suppl_supplementary_appendix_s1.docx]

**Supplementary Appendix S1**

*DNA extraction and library preparation*

Between 600-800 µl Saliva sample was mixed with 850 µl phosphate-buffered saline and heated up to 95⁰C for 15 minutes before centrifugation. The saliva samples were centrifuged at 10,000 rpm for 2 minutes. The pellet was used for DNA extraction by adding 700 µl SL1 and 150 µl Enhancer SX buffers to the pellet, transfer the mix to a bead tube and follow the manufactory protocol for the Machery Nagel Nucleospin Soil Kit. The purified DNA underwent a two-step PCR where 10.4µl PCR grade water, 4 µl Phusion HF Buffer, 200 µM dNTP mix, 0.02 U/µl Phusion Hot Start II DNA Polymerase 0.5 µM forward primer (341.2FDI 5'-ACACTCTTTCCCTACACGACGCTCTTCCGATCTCCTACGGGNGGCWGCAG-3), 0.5 µM reverse primer (805.2RDI 5'-AGACGTGTGCTCTTCCGATCTGACTACHVGGGTATCTAATCC-3') and 3 µl DNA template was mixed in a total of 20 µl. The PCR program was with an initial denaturation at 95⁰C for 3 min followed by 15 cycles of denaturation at 95⁰C for 30 s, annealing at 55⁰C for 30 s and elongation at 72⁰C for 30 s. The end elongation step was at 72⁰C for 3 min followed by a hold at 4⁰C. The second PCR mix was 12.4 µl PCR grade water, 4 µl Phusion HF Buffer, 200 µM dNTP mix, 0.02 U/ µl Phusion Hot Start II DNA Polymerase, 0.5 µM forward primer (P2F# 5'-AATGATACGGCGACCACCGAGATCTACAC-8-N barcode-ACACTCTTTCCCTACACGACG-3'), 0.5 µM reverse primer (P2R# 5'-CAAGCAGAAGACGGCATACGAGAT- 8-N barcode -GTGACTGGAGTTCAGACGTGTGCTCTTCCGATCT-3') and 1 µl DNA template. The PCR product was purified using AMPure Beads in 0.9 x ratio and the samples were pooled in equimolar amounts. The sequencing was done on Illumina MiSeq in 300 bp paired end mode.

*Bioinformatics Analysis*

The raw fastq files were processed into a zero radius OTU count table using the USEARCH pipeline (Edgar, 2013). The 300 bp paired end reads were merged into a single amplicon fragment using fastq_mergepairs-relabel @, the universal primer sequences are trimmed using fastx_truncate-stripleft 15 -stripright 21 and low quality sequences were filtered using fastq_filter-fastq_maxee 1. Unique amplicons were identified using fastx_uniques -relabel Uniq-sizeout. To filter out potential chimeric amplicons unoise3 was used and usearch_global-strand plus -id 0.97 was used to remap the raw reads to the filtered unique amplicons. The taxonomic classification of the unique filtered amplicons were conducted with the QIIME feature-classifier classify-sklearn [Bolyen et al. 2019] and the Human Oral Microbiome Database v15.22 [Chen et al. 2010].

*References*

Bolyen E, Rideout JR, Dillon MR *et al.* Reproducible, interactive, scalable and extensible microbiome data science using QIIME 2. *Nat Biotechnol.* 2019;37;852–857.

Chen T, Yu WH, Izard J, Baranova OV, Lakshmanan A, Dewhirst FE. The Human Oral Microbiome Database: a web accessible resource for investigating oral microbe taxonomic and genomic information. Database (Oxford). 2010 Jul 6;2010:baq013.
